# Plant Cryptochromes Illuminated: A Spectroscopic Perspective on the Mechanism

**DOI:** 10.3389/fchem.2021.780199

**Published:** 2021-11-24

**Authors:** Lukas Goett-Zink, Tilman Kottke

**Affiliations:** ^1^ Department of Chemistry, Bielefeld University, Bielefeld, Germany; ^2^ Biophysical Chemistry and Diagnostics, Medical School OWL, Bielefeld University, Bielefeld, Germany

**Keywords:** photoreceptor, photolyase, in-cell spectroscopy, flavin, UV-vis spectroscopy, EPR (electron paramagnetic resonance), FTIR (Fourier transform infrared spectroscopy), blue light receptor

## Abstract

Plant cryptochromes are central blue light receptors for the control of land plant and algal development including the circadian clock and the cell cycle. Cryptochromes share a photolyase homology region with about 500 amino acids and bind the chromophore flavin adenine dinucleotide. Characteristic for plant cryptochromes is a conserved aspartic acid close to flavin and an exceptionally long C-terminal extension. The mechanism of activation by excitation and reduction of the chromophore flavin adenine dinucleotide has been controversially discussed for many years. Various spectroscopic techniques have contributed to our understanding of plant cryptochromes by providing high time resolution, ambient conditions and even in-cell approaches. As a result, unifying and differing aspects of photoreaction and signal propagation have been revealed in comparison to members from other cryptochrome subfamilies. Here, we review the insight from spectroscopy on the flavin photoreaction in plant cryptochromes and present the current models on the signal propagation from flavin reduction to dissociation of the C-terminal extension.

## Introduction

**FIGURE 1:**
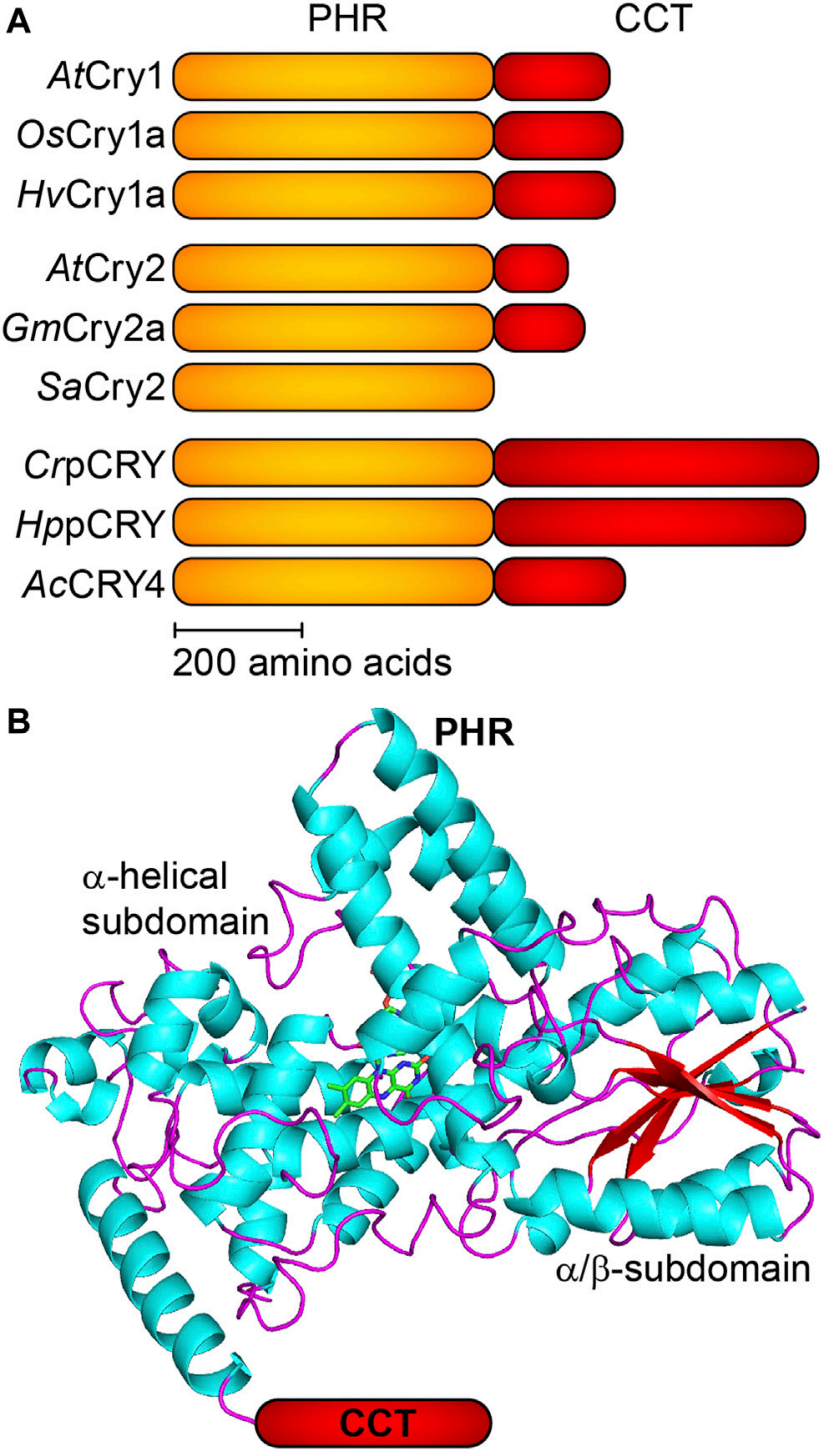
Schematic domain topology and structure of plant cryptochromes (pCRY). **(A)** Plant cryptochromes share a conserved PHR domain and a CCT of varying length. *Cr*pCRY exhibits the longest CCT with ∼500 amino acids. The selected pCRY originate from *Arabidopsis thaliana* (*At*CRY1 and *At*CRY2), rice *Oryza sativa* (*Os*CRY1a), barley *Hordeum vulgare* (*Hv*CRY1a), soybean *Glycine max* (*Gm*CRY2a), white mustard *Sinapis alba* (*SaCRY2*), green alga *Chlamydomonas reinhardtii* (*Cr*pCRY), green alga *Haematococcus pluvialis* (*Hp*pCRY) and fern *Adiantum capillus-veneris* (*Ac*CRY4). **(B)** The PHR domain of pCRY comprises an α-helical subdomain binding FAD and ATP as well as an α/β-subdomain with a parallel β-sheet (PDB:1U3D). The CCT is connected to the C-terminal region of the PHR but is not resolved in the crystal structure.

Plant cryptochromes (pCRY) are a subfamily of the large cryptochrome/photolyase superfamily (CPF) of photoreceptors, DNA repair enzymes and clock proteins (Chaves et al., 2011). pCRY share a 500 amino acid photolyase homology region (PHR) and differ in the length of the unconserved C-terminal extension (CCT) with little structural elements (Figure 1). Flavin adenine dinucleotide (FAD) is bound noncovalently as a chromophore to the FAD binding pocket of the PHR. pCRY regulate many photomorphogenetic responses such as the flowering time as well as the determination of the day length in land plants and the cell cycle in green algae (Wang et al., 2014; Kottke et al., 2017).

The homology in sequence and structure of pCRY to cyclobutane pyrimidine dimer (CPD) photolyases might suggest that they share a common mechanism. However, extensive spectroscopic studies have shown that the mechanisms of these two protein subfamilies are distinct. A key difference between CPD photolyases and pCRY is the initial state of the cofactor. CPD photolyases bind fully reduced FAD (FADH^−^) as the dark form prior to catalysis, which is formed by a so-called photoactivation reaction from the precursors oxidized FAD (FAD_ox_) and FAD neutral radical (FADH•) (Sancar, 2003). In contrast, the dark form of pCRY is FAD_ox_, which has been confirmed by a series of studies *in vitro* and in cells (Banerjee et al., 2007; Bouly et al., 2007; Engelhard et al., 2014; Goett-Zink et al., 2021). Still, some unifying aspects have been found between the photoactivation reaction of CPD photolyase and the photoreaction of FAD_ox_ in pCRY.

It should be noted that spectroscopic studies have almost exclusively been performed on only two members of the pCRY family, cryptochrome 1 from *Arabidopsis thaliana* (*At*CRY1) (Lin et al., 1995) and plant cryptochrome from *Chlamydomonas reinhardtii* (*Cr*pCRY or CPH1) (Reisdorph and Small, 2004)*.* Issues with stability and solubility of the full-length proteins limited these investigations mostly to the PHR, neglecting the influence of the CCT. In the following, pCRY will be used synonymously to pCRY-PHR and the few studies on the full-length proteins will be highlighted.

Here, we will discuss the initial steps in the photoreaction of pCRY along with similarities and differences to CPD photolyases. Moreover, we will reveal the current status of insight into the subsequent signal propagation from the chromophore through the PHR to the CCT. Last, we will address key differences in mechanism to other members of the cryptochrome superfamily and give an outlook on open questions with respect to the light-induced clustering of the PHR.

## Formation of the Flavin Neutral Radical Studied by Time-Resolved Approaches

**FIGURE 2:**
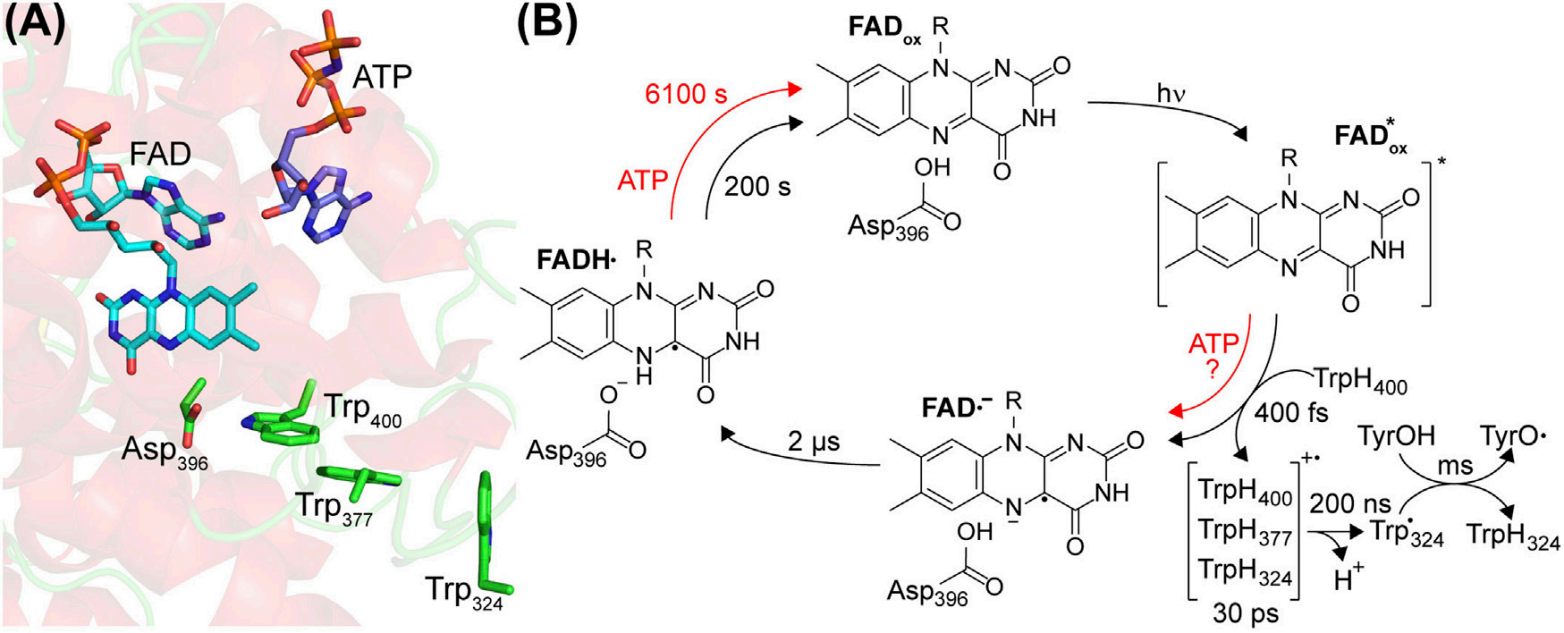
Center of the photoreaction of plant cryptochromes and photocycle of FAD. **(A)** The α-helical domain of the PHR binds oxidized FAD and ATP. Close to the FAD, a tryptophan-triad Trp_400_, Trp_377_ and Trp_324_ acts as electron donor and aspartic acid Asp_396_ acts as proton donor (according to *At*CRY1 numbering). **(B)** FAD_ox_ is excited by UV-A/blue light initiating an ultrafast electron transfer from Trp_400_. In the presence of ATP, an alternative electron pathway has been proposed. The resulting FAD anion radical (FAD•^−^) is stabilized by electron hopping events in the tryptophan triad and deprotonation of TrpH_324_•^+^ to the bulk. Subsequently, Asp_396_ protonates FAD•^−^ to the FAD neutral radical (FADH•), which represents the signaling state. The formation of a tyrosyl radical (TyrO•) takes place in the millisecond time region. Binding of ATP enhances the yield of the photoreaction and decelerates the reoxidation to FAD_ox_ as indicated by the time constants determined for *Cr*pCRY.

Light absorption in pCRY is dominated by the chromophore FAD_ox_. An antenna molecule is not bound to pCRY, at least not stoichiometrically (Immeln et al., 2007; Hoang et al., 2008), in contrast to the binding of the antenna 5,10-methenyltetrahydrofolic acid (MTHF) to CPD photolyase (Jorns et al., 1984). This difference might be rationalized by the much higher extinction coefficient of FAD_ox_ in pCRY than of FADH^−^ in CPD photolyase. The crystal structure of pCRY shows side chains filling the binding pocket that might lower the affinity to MTHF (Brautigam et al., 2004). Instead, pCRY bind adenosine triphosphate (ATP) (Bouly et al., 2003) and other nucleotides (Engelhard et al., 2014), most likely in the access cavity close to the chromophore, which is in DNA photolyase responsible for binding of damaged DNA (Figure 2A) (Brautigam et al., 2004; Ma et al., 2020).

The FAD_ox_ in pCRY absorbs in the UVA and blue spectral region up to λ ∼ 500 nm resulting in a loss of FAD_ox_ and the formation of FADH• (Lin et al., 1995; Giovani et al., 2003). This photoreaction in pCRY was studied at high time resolution by femtosecond broadband transient UV-vis spectroscopy. With a time constant of 400 fs, an electron transfer takes place from the nearby tryptophan (TrpH_400_) to the excited FAD_ox_* forming the flavin anion radical (FAD•^−^) and the corresponding tryptophan cation radical (TrpH_400_•^+^) (Figure 2B) (Immeln et al., 2012). The redox potentials for reduction of FAD_ox_ to FADH• in pCRY and oxidation of TrpH to (Trp•,H^+^) in solution are −153 mV (Balland et al., 2009) and 1.00 V (Mahmoudi et al., 2016), respectively, precluding reduction of FAD_ox_ in the dark. Excitation results in configurations with similar energy of FAD_ox_* and the charge transfer state TrpH_400_ to FAD_ox_* (Cailliez et al., 2014). The FAD•^−^ is stabilized against recombination by a tryptophan triad, which increases the distance between the cation and anion radicals by electron hopping between TrpH_400_, TrpH_377_ and TrpH_324_ (or alternatively TrpH_379_) within 30 ps (Immeln et al., 2012). This triad is conserved in the CPF family and the respective electron transfer processes have been studied in detail for photoactivation of CPD photolyase, albeit starting with excitation of FADH• (Aubert et al., 2000). In the next step, TrpH•^+^ in pCRY deprotonates to Trp• with τ = 200 ns, most likely releasing the proton to the bulk (Müller et al., 2014).

Time-resolved UV-vis spectroscopy revealed that FAD•^−^ is protonated to give FADH• with a time constant of 2 µs (Figure 2B) (Langenbacher et al., 2009; Maeda et al., 2012). The FADH• is considered to be the signaling state in pCRY. Infrared difference spectroscopy, in particular the time-resolved step-scan technique, was used to identify the proton donor as the conserved Asp_396_ (Kottke et al., 2006; Hense et al., 2015; Thöing et al., 2015), which is fully protonated in the dark at physiological pH (Müller et al., 2014; Schroeder et al., 2018). Accordingly, proton transfer is completely decoupled from the electron transfer, which was confirmed by a quantum mechanical molecular dynamics approach (Lüdemann et al., 2015). The presence of the intrinsic proton donor Asp_396_ close to FAD is one of the major differences to CPD photolyase, which contains Asn at this position (Figure 2).

As final electron transfer step, Trp• reacts with a surface-exposed tyrosine to a tyrosine radical (TyrO•) with τ = 1 ms in full-length pCRY (Giovani et al., 2003), which is then reduced in the millisecond time range by the bulk, strongly depending on the concentration of external reductant and on the pCRY member (Giovani et al., 2003; Thöing et al., 2015). Interestingly, the identification of this tyrosine in pCRY is lacking, whereas in other cryptochromes specific tyrosines have been identified (Oldemeyer et al., 2016; Zoltowski et al., 2019).

The quantum yield of FADH• formation is low with only 2% (Giovani et al., 2003), but can be significantly increased by the addition of ATP and reductant to ∼14% for *At*CRY1 (Müller et al., 2014). The increased FADH• formation has been attributed to structural changes in pCRY caused by the binding of ATP (Iwata et al., 2020), which leads to a closer contact of FAD and Trp_400_ enhancing the electron transfer (Cailliez et al., 2014). This quantum yield is still comparatively low for a photoreceptor pointing to several loss processes, considering that other cryptochromes have shown quantum yields of up to 43% (Zoltowski et al., 2019). Moreover, the effect of ATP is less pronounced in *Cr*pCRY in the absence of reductant, for which only 2% of the absorbed photons lead to a stable product on the minute time range (Schroeder et al., 2018). Interestingly, experiments on pCRY demonstrated a sensitivity in yield of FADH• on the external magnetic field acting on the singlet/triplet interconversion of the radical pair FAD•^−^/Trp•^+^ (Maeda et al., 2012), which inspired further investigations on the role of cryptochromes in magnetoreception.

FADH• is strongly stabilized by protonation (Hense et al., 2015), by reduction of Trp• (Müller et al., 2014) and by the presence of ATP (Immeln et al., 2007; Hense et al., 2015). Accordingly, dependent on the specific pCRY member and on the buffer conditions, the reoxidation to FAD_ox_ by oxygen takes a few minutes to hours at room temperature (Figure 2B). Important insight was provided by the finding in a screen that two point mutations distant from the FAD binding pocket at helix α13 strongly modulate the recovery time of FAD_ox_ (Taslimi et al., 2016), which might be related to the signaling mechanism. A competing pathway for reoxidation requires the presence of high light intensity and strong reductant to produce FADH^−^, which then readily reacts with oxygen (Müller and Ahmad, 2011).

## In-Cell Spectroscopic Approaches Contribute to Our Understanding of the Mechanism

The formation of FADH• from FAD_ox_ in the photoreaction of pCRY was confirmed by in-cell fluorescence and in particular by in-cell electron paramagnetic resonance (EPR) spectroscopy on frozen insect cells (Banerjee et al., 2007; Bouly et al., 2007). The decay of FADH• after illumination was slowed down in living *E. coli* cells to a similar extent as in the presence of ATP *in vitro*, as shown by in-cell UV-vis spectroscopy (Goett-Zink et al., 2021). Importantly, the decay of EPR signals in insect cells agreed with the time range of the physiological response, further supporting the role of FADH• as signaling state (Herbel et al., 2013). However, the role of the conserved tryptophan triad as an essential part of the photoreaction has been controversial (Ahmad, 2016), because single point mutations in the tryptophan triad of pCRY *in planta* did not abolish the physiological response (Li et al., 2011; Gao et al., 2015). Instead, the tryptophan triad might play a role in the structural integrity because some of these mutants show constitutively active phenotypes (Li et al., 2011; Gao et al., 2015). Photoreduction of FAD_ox_ in pCRY in insect cells similarly proceeded despite such point mutations (Engelhard et al., 2014). Moreover, small metabolites*,* in particular ATP, enhanced the photoreaction also with mutations in the tryptophan triad (Engelhard et al., 2014). It should be noted that a role of ATP as a reducing agent can be excluded. Therefore, an alternative electron pathway in the presence of cellular nucleotides was proposed, which is independent of the tryptophan triad. All these observations in cells have changed the view on the activation mechanism of pCRY *in vivo*.

## Signal Propagation in the Receptor From Flavin Adenine Dinucleotide to C-Terminal Extension—Insight From Spectroscopy

**FIGURE 3:**
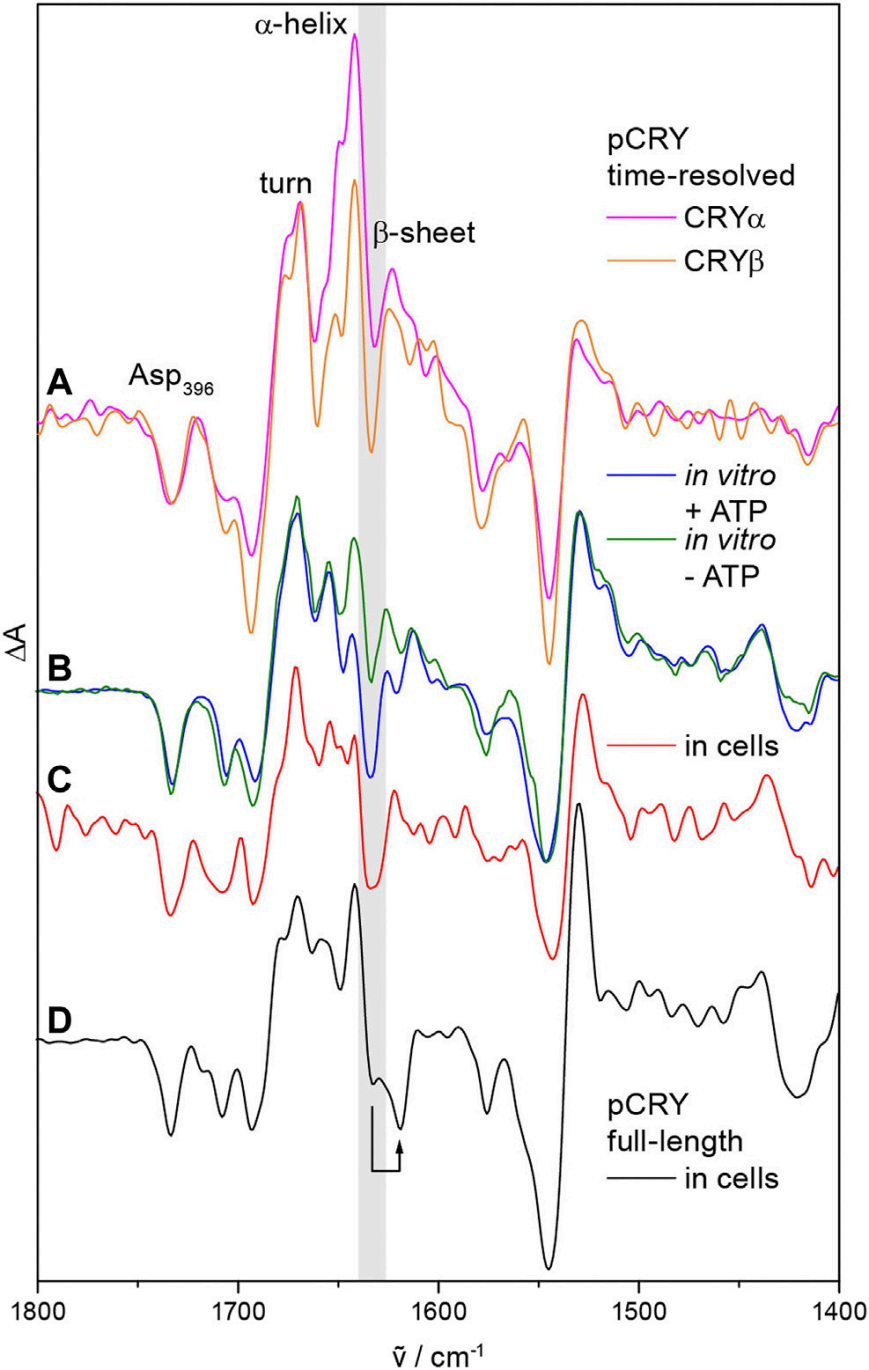
Conformational changes in pCRY observed by time-resolved, static and in-cell infrared difference spectroscopy. **(A)** Time resolved step-scan experiments on the PHR domain detected an increase in α-helical and turn elements, which is characteristic for the intermediate CRYα with a lifetime of 500 µs. Subsequently, a loss of β-sheet content is detected representing the intermediate CRYβ (highlighted in gray) with a lifetime of 29 ms. **(B)** The presence of ATP stabilizes the CRYβ intermediate *in vitro* into the time range of minutes as observed by static experiments. **(C)** Similarly, cellular nucleotides stabilize CRYβ in bacterial cells as determined by in-cell spectroscopy. **(D)** Full-length pCRY shows a shift of the β-sheet signal attributed to the association of the CCT close to the β-sheet of the PHR as shown by in-cell spectroscopy. Spectra were taken from (Thöing et al., 2015; Schroeder et al., 2018; Goett-Zink et al., 2021).

pCRY responds with conformational changes to the photoreduction of FAD_ox_. Time-resolved step-scan and rapid-scan infrared difference spectroscopy identified two major intermediates after light-induced activation of the PHR, CRYα and CRYβ (Figure 3A) (Thöing et al., 2015). Changes in α-helical elements and turn structures are detected already few microseconds after excitation representing the CRYα intermediate. Subsequently, a loss of β-sheet content takes place with a time constant of 500 µs, which is characteristic for CRYβ and occurs concomitant with the formation of TyrO•. It should be highlighted that the only β-sheet of pCRY is present in the α/β-subdomain at a distance of ∼25 Å to FAD (Figure 1B) (Brautigam et al., 2004). This assignment indicates that the signal propagates from FADH• to this parallel β-sheet leading to a reorganization of the β-sheet rather than an unfolding. The presence of ATP stabilizes the conformational changes of CRYβ *in vitro* (Figure 3B) and in bacterial cells (Figure 3C) from a transient species with τ = 29 ms into the minute time range (Schroeder et al., 2018; Goett-Zink et al., 2021). Both, *At*CRY1 and *Cr*pCRY showed such stabilization of conformational changes in the presence of ATP (Schroeder et al., 2018; Iwata et al., 2020) suggesting that CRYβ is a key component in the signal progression of pCRY.

The isolated CCT of pCRY is largely unstructured as found by circular dichroism and nuclear magnetic resonance (NMR) spectroscopy (Partch et al., 2005). Recent studies on full-length pCRY in bacterial cells by in-cell infrared difference spectroscopy showed an association of the CCT close to the β-sheet of the PHR in the dark, thereby downshifting the signal of the β-sheet (Figure 3D) (Goett-Zink et al., 2021). Upon illumination, the CCT dissociates from the PHR and increases the diffusion coefficient with τ = 400 ms as demonstrated by transient grating spectroscopy on full-length pCRY (Kondoh et al., 2011). The dissociation increases the accessibility of the CCT to proteolytic digestion *in vitro* (Partch et al., 2005). Together, spectroscopic studies on pCRY indicate that the light-induced signal propagates from FAD to the β-sheet with bound CCT resulting in a β-sheet reorganization, which then induces the dissociation of the CCT from the PHR (Goett-Zink et al., 2021).

The initiating step of the signal propagation from the chromophore to the protein is not fully understood. A model has been proposed in which the negative charge of deprotonated Asp_396_ repels bound negatively charged ATP and leads to a dissociation of the CCT covering the ATP binding site (Müller and Bouly, 2014). Direct experimental evidence for a light-induced release of ATP or a coverage of the ATP binding site by the CCT is lacking. It is challenging to design studies on pCRY mutants without a negative charge in the FAD binding pocket, because the exchange of Asp_396_ to Asn and Cys leads to the light-induced formation of charged FADH^−^ and FAD•^−^, respectively (Burney et al., 2012; Hense et al., 2015). Infrared spectroscopic studies on pCRY lacking Asp_396_ (in the D396C mutant) showed that FAD•^−^ is already sufficient to induce β-sheet reorganization in pCRY, albeit the formation of FADH• and/or deprotonated Asp_396_ stabilizes the conformational changes into a physiological relevant time region (Hense et al., 2015; Schroeder et al., 2018). Furthermore, time resolution has not been sufficient yet to link the formation of the intermediate CRYα to either formation of FAD•^−^/Asp_396_OH or FADH•/Asp_396_O^−^. Hence, electrostatic interactions/repulsion exerted by FAD•^−^ and deprotonated Asp_396_, respectively, are likely key components of signal propagation in pCRY. Similar mechanisms have been proposed to be active in *Drosophila* cryptochrome by the negative charge of FAD•^−^ (Ganguly et al., 2016) as well as in other photoreceptor families such as photoactive yellow proteins (Kottke et al., 2018).

## Similarities and Key Differences in Mechanism Compared to Other Cryptochrome Subfamilies

A unifying aspect of the mechanism of pCRY valid also for other cryptochrome subfamilies is the decoupled electron and proton transfer to FAD_ox_. As a result, FAD•^−^ is formed and stabilized to a different extent. pCRY is differentiated from other cryptochrome subfamilies by the conserved Asp_396_ as an intrinsic proton donor to FAD•^−^ within few microseconds. In other cryptochromes such as CRY-DASH, animal and animal-like cryptochromes the Asp_396_ is exchanged by an asparagine (Brudler et al., 2003). These cryptochromes show a millisecond protonation of FAD•^−^, most likely from the bulk (Lacombat et al., 2019). Different extents of stabilization have been reported within the DASH subfamily (Iwata et al., 2010). For insect cryptochromes, a cysteine is conserved at this position and FAD•^−^ is stable for minutes (Berndt et al., 2007; Zoltowski et al., 2011). It should be noted that a single exchange of cysteine for aspartate in insect cryptochromes led to formation of a neutral radical (Öztürk et al., 2008), albeit not with the characteristic blue shift of the FADH• absorbance bands in pCRY attributed to the charge of deprotonated Asp_396_ close to FADH• (Immeln et al., 2010). These observations show that a specific, hydrophobic environment in the FAD binding pocket of pCRY generates a protonated Asp_396_ in the dark (Kottke et al., 2006). The full protonation of Asp_396_ is aided by the binding of ATP, which results in an upshift of the pK_a_ value (Müller et al., 2014; Iwata et al., 2020).

Asp_396_ in pCRY not only acts as proton donor, but influences the redox potential of the FAD. The potential for reduction of FADH• to FADH^−^ is lowered as compared to CPD photolyase with an Asn conserved at this position (Balland et al., 2009), supported by the finding that the D396N mutant of pCRY forms FADH^−^ instead of FADH• after illumination (Burney et al., 2012). Moreover, replacement of Asn by Asp in CPD photolyase leads to stabilization of FAD_ox_ instead of FADH• in the dark *in vitro*, whereas hydrogen bonding and the protein environment for FAD are quite similar (Damiani et al., 2011). Accordingly, pCRY is primed to bind FAD_ox_ as the dark form for blue light reception. In contrast, animal-like cryptochromes have been claimed to bind FADH• as the dark form for white light reception (Beel et al., 2012), aided by a very efficient photoreduction from FAD_ox_ (Lacombat et al., 2019). Recently, CRY-DASH have been postulated to bind FADH^−^ in the dark for UVA reception (Rredhi et al., 2021).

In other members of the CPF than pCRY, an antenna chromophore is additionally associated in a stoichiometric ratio such as 5,10-methenyltetrahydrofolic acid (MTHF) to CRY-DASH or 8-hydroxy-deazaflavin (8-HDF) to animal-like cryptochromes (Song et al., 2006; Franz et al., 2018). These antenna molecules aid in increasing the extinction coefficient for excitation, which might be rationalized by the lower extinction coefficient of FADH• or FADH^−^ as compared to FAD_ox_.

Another property distinguishing pCRY from other cryptochromes is the exceptional length of the CCT for most of the pCRY members (Figure 1A). This difference needs to be considered in the comparison of mechanisms to *Drosophila* CRY with a CCT of only 23 amino acids or to CRY-DASH, for which some fungal members have CCT with up to 200 amino acids (Froehlich et al., 2010). Nevertheless, as for pCRY, undocking of the CCT from the PHR after photoactivation was demonstrated in *Drosophila* CRY (Chandrasekaran et al., 2021). Interestingly, *Sinapis alba* pCRY does not contain a CCT, which poses some questions on its signal transduction mechanism (Malhotra et al., 1995).

## Outlook on the Molecular Basis of Clustering

Several open questions in the mechanism of pCRY have been highlighted in the previous sections. A further fascinating aspect of pCRY is the light-induced homooligomerization and formation of photobodies as observed with phytochrome B in *A. thaliana* (Mas et al., 2000; Bugaj et al., 2013). Blue-light illumination of pCRY induces clustering via PHR, which is essential for the function in *A. thaliana* (Wang and Lin, 2020). The photooligomerization of PHR has been successfully established on *At*CRY2 and applied in optogenetic tools for light-induced activation of effector proteins (Bugaj et al., 2013; Taslimi et al., 2014). Illuminated *At*CRY2 forms tetrameric units of the PHR in a “doughnut” shaped structure with interaction sites at the α/β-subdomain and the C-terminal region of the α-helical subdomain as observed by cryogenic electron microscopy (Ma et al., 2020). The introduction of several single point mutations in the α-domain of *At*CRY2 abolished homooligomerization, whereas a E490G mutation shows increased oligomerization properties (Taslimi et al., 2014; Ma et al., 2020). Albeit these observations indicate the involvement of these amino acids in oligomerization, the molecular basis of clustering in pCRY is not yet understood. Time-resolved spectroscopic methods for studying structural changes on pCRY with single point mutations might address these unresolved questions.
